# P-1498. Real-World Vaccine Effectiveness and Impact of the 4CMenB Vaccine in Infants, Children, and Adolescents: A Systematic Literature Review

**DOI:** 10.1093/ofid/ofaf695.1682

**Published:** 2026-01-11

**Authors:** Pavo Marijic, Lucian Gaianu, Gaurav Mathur, Thatiana Pinto, Anar Andani, Reena Ladak, Elise Kuylen, Karolina Szewczyk, Elzbieta Olewinska, Beata Smela, Helen Petousis-Harris, Zeki Kocaata

**Affiliations:** GSK, Munich, Bayern, Germany; GSK, Munich, Bayern, Germany; GSK, Philadelphia, Pennsylvania, USA, Philadelphia, Pennsylvania; GSK, Munich, Bayern, Germany; GSK, Munich, Bayern, Germany; GSK, Philadelphia, Pennsylvania, USA, Philadelphia, Pennsylvania; GSK, Munich, Bayern, Germany; Clever-Access, Kraków, Malopolskie, Poland; Clever-Access, Kraków, Malopolskie, Poland; Clever-Access, Kraków, Malopolskie, Poland; The University of Auckland, Auckland, Auckland, New Zealand; GSK, Munich, Bayern, Germany

## Abstract

**Background:**

Meningococcal serogroup B (MenB) is a leading cause of invasive meningococcal disease. The multicomponent MenB vaccine (4CMenB) has been widely used since 2013. To establish a robust range of vaccine effectiveness (VE) and determine the vaccine impact (VI) of 4CMenB programs on MenB incidence in infants, children, and adolescents, a systematic literature review of real-world evidence was conducted.

**Figure 1.**
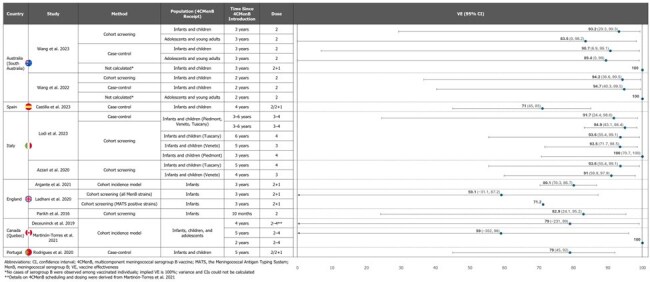
4CMenB Vaccine Effectiveness Against MenB in Fully Vaccinated Infants, Children, Adolescents, and Young Adults

**Table 1.**
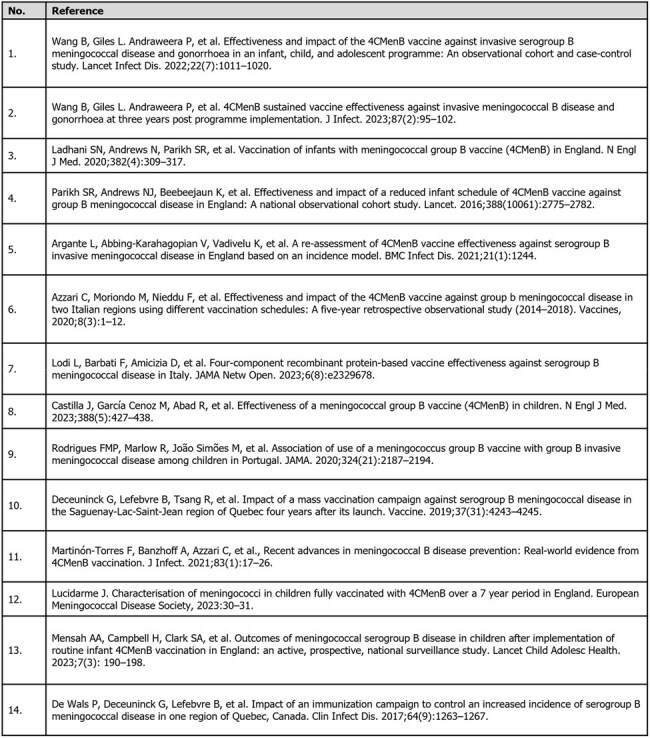
Study References for Vaccine Effectiveness and Impact

**Methods:**

MEDLINE, Embase, gray literature, and clinical trial registries were searched in November 2024; studies were screened against preset criteria per PRISMA guidelines. VE and VI were synthesized separately for infants, children, and adolescents/young adults.

**Results:**

Nine studies from Australia, Canada, England, Italy, Portugal, and Spain evaluated VE; all included infants, 8 included children, 2 included adolescents.^1–11^ Nine studies from Australia, Canada, England, and Italy reported VI.^1–7, 10–14^ (Table 1)

High estimated VE against MenB was reported for fully vaccinated (2–4 doses) infants and children. In South Australia (SA), VE was 90.7–94.7% in infants/children with 2 doses 2–3 years (y) after introducing a 4CMenB program and no cases occurred in those with 3 doses after 3 y.^1,2^ In England, VE was 82.9% 10 months (m) after 4CMenB introduction in infants and 59.1–80.1% after 3 y in infants/children.^3–5^ VE across Italy ranged 91.0–100% 3–6 y after 4CMenB introduction in infants/children.^6,7^ In Spain, VE was 71% in infants/children 4 y after 4CMenB became available.^8^ In Portugal, VE was 79% in infants/children 5 y after 4CMenB was licensed.^9^ (Figure 1)

VE was also high in fully vaccinated (2 doses) adolescents/young adults. In SA, VE was 100% 2 y after 4CMenB program introduction and 83.5–89.4% after 3 y.^1,2^ In Quebec, after a 4CMenB campaign among those aged 2 m–20 y, VE was 100%, 79%, and 59%, at 2, 4, and 5 y, respectively.^10,11,14^

VI (decrease in MenB incidence post-4CMenB program) in targeted age groups was 75% in England (18 weeks [w]–2 y) after 3 y, 63% and 79% in SA (12 w–11 m and adolescents) after 3 y, 50% across Italy (0–6 y) after 6 y, and 94% in Quebec (2 m–20 y) after 5 y. ^1–7, 10–14^

**Conclusion:**

With 11 years of global use, 4CMenB has demonstrated robust real-world VE (59–100%) and substantial VI in reducing MenB incidence in infants, children, and adolescents.

Funding: GSK VEO-001056

**Disclosures:**

Pavo Marijic, PhD, GSK: employee|GSK: Stocks/Bonds (Public Company) Lucian Gaianu, MSc, GSK: Employee Gaurav Mathur, MD, GSK: Employee|GSK: Stocks/Bonds (Public Company) Thatiana Pinto, PhD, GSK: employee|GSK: Stocks/Bonds (Public Company) Anar Andani, BSc, Medical director, GSK: Employee|GSK: Stocks/Bonds (Public Company) Reena Ladak, MS, GSK: Employee Elise Kuylen, PhD, GSK: Employee|GSK: Stocks/Bonds (Public Company) Karolina Szewczyk, MSc, GSK: Employee of Clever-Access, which was paid by GSK to conduct this study Elzbieta Olewinska, MSc, GSK: Employee of Clever-Access, which was paid by GSK to conduct this study Beata Smela, PhD, GSK: Employee of Clever-Access, which was paid by GSK to conduct this study Helen Petousis-Harris, PhD, Bexsero and gonorrhea trial (USA): Data and Safety Monitory Board Member|CDC: Funding to institution|GSK: Advisor/Consultant|GSK: Funding to institution; payment for study|Maternal pneumococcal vaccine trial (Australia): Data and Safety Monitory Board Member|New Zealand Medical Council: Advisor/Consultant|The Ministry of Health New Zealand: Funding to institution Zeki Kocaata, PhD, GSK: Employee|GSK: Stocks/Bonds (Public Company)

